# Supplementary material for: Come together: A unified description of the escalator capacity
Source: PLoS One. 2023 Mar 6;18(3):e0282599. doi: 10.1371/journal.pone.0282599 (PMC9987794; doi:10.1371/journal.pone.0282599)
Supplement: S1 Appendix — (PDF) [file pone.0282599.s001.pdf]

Appendix: Possible speed dependence of the time gap

In case we interpret T as reaction time of entering passengers, it can be assumed that the T-value increases if the escalator is operated at higher speeds. The reason is that the adaptation process of the walking agent to adapt to the fast-moving escalator is more complicated and, therefore, it is likely that passengers will hesitate longer when entering a faster escalator than a slower one. Unfortunately, and to our best knowledge, reliable empirical data regarding an individual and speed-dependent hesitation time of passengers entering escalators are lacking. Regardless, however, in the obtained capacity formula (16), a speed-dependent hesitation time can be taken into account by adding terms to the finite time gap T, e. g. in form of a power series

$$T \;\rightarrow\; \underbrace{T}_{\text{reaction time}} \;+\; \underbrace{A_1 \cdot v_{\text{esc}}^1 + A_2 \cdot v_{\text{esc}}^2 + \dots}_{\text{hesitation time}} \,. \tag{18}$$

The implementation of speed-dependent terms in Eq (18) is by no means unique since the underlying velocity-dependence is (so far) unknown. Using a power series is only *one possible* realization. The exact values of the introduced coefficients A_i are (of course) unknown and have to be determined e. g. through dedicated experiments or field studies. Regarding in particular capacity values stated in [3, 5, 19], however, good agreement is achieved by using $A_5 = 0.6\,\text{s}^6\text{m}^{-5}$ and $A_j = 0$ for the other coefficients, see Fig 10.

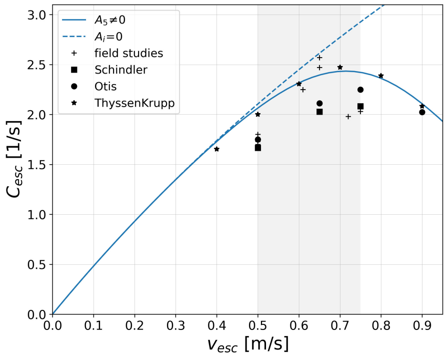

Fig 10. Speed-dependent capacity of an escalator in case of a speed-dependent hesitation time. The capacity function is obtained replacing $T \rightarrow 0.15\,\text{s} + 0.6\,\text{s}^6\text{m}^{-5} \cdot v_{\text{esc}}^5$ in Eq (16). The escalator parameters are fixed by $d_{\text{step}} = 0.4\,\text{m}$ and $w = 1\,\text{m}$ ($\mathcal{O}_0 = 2$).

As can be seen, introducing a finite hesitation time, it is even possible to model the case that the capacity of an escalator *decreases* for larger conveyor speeds as stated e. g. by [3, 5, 19]. Using Eq (16) together with replacement (18), the decrease can be implemented such that it does not significantly affect the range at which escalators are most commonly operated, namely $v_{\text{esc}} \leq 0.5\,\text{m}\,\text{s}^{-1}$. Further statements regarding a speed-dependent hesitation time related to the entering process at escalator are difficult to make due to missing comparison possibilities. Both the collection of corresponding empirical data, e. g. through dedicated experiments and field studies, as well as the modeling of the latter therefore remain tasks for future work.

References

1. OTIS. Planungshandbuch - Projektierung von Fahrtreppen und Fahrsteigen; 2022.
2. ThyssenKrupp Elevator Americas. Escalators & Moving Walks planning guide; 2013.
3. Majo AJ. A study of escalators and associated flow systems. MSc Degree Report; 1966.
