# Supplementary material for: Come together: A unified description of the escalator capacity
Source: PLoS One. 2023 Mar 6;18(3):e0282599. doi: 10.1371/journal.pone.0282599 (PMC9987794; doi:10.1371/journal.pone.0282599)
Supplement: Attachment — Submitted filename: Response to Reviewers.pdf [file pone.0282599.s002.pdf]

Response to the comments of reviewer 1

Dear reviewer,

we would like to thank you for your efforts in studying our article, and your useful suggestions, which contribute to improve our work. Please find below our answers to each of your comments and a description of the corresponding modifications done in the manuscript.

The authors studied the dynamics of passengers on escalators through the perspective of numerical simulation. They derived a space-continuous model to describe how agents change from walking on the plain to standing on the escalator. They also deduced a capacity formula that is in agreement with results found in literature. Finally, they claim that the deduced capacity formula allows one to find an optimal conveyor speed simultaneously compatible with safety, comfort, and performance demands. Overall the manuscript is well written and organized. It brings contributions to the understanding of the dynamics of passengers on escalators, and it seems appropriate for PLOS ONE. However, I have some questions that I would like addressed:

Point 1 : In section "Simulation of escalators", the authors describe some parameters used in the simulations. Precisely, in the last paragraph the authors write "we ensure that the relation $\alpha > \beta$ holds in all simulations, where α is the incoming agent flow and β is the flow on the escalator." Do the authors believe that the α/β ratio could affect the results? Why or why not?

Response 1 : In the present contribution we investigate the handling capacity of an escalator which is the *maximum possible* flow β that can be realized over an extended period of time. Using simulations, this is the quantity that we measure. The incoming agent flow α, on the other hand, can be freely adjusted in the simulations and the question arises how this should be done. Regarding a stationary state, two system states are possible:

1.) Free state $\alpha = \beta$, (incoming agent flow in front is the same as the flow on the escalator). This is the normal operational mode of an escalator. Each incoming agent has directly access to the escalator and no congestion is forming in front of the facility. Since we are looking for the *maximum possible* value of β, however, it is not clear that β indeed coincides with the capacity $C_{esc} = \max(\beta)$.

2.) Congested state $\alpha > \beta$, (incoming agent flow in front is larger than the flow on the escalator). This is the capacity mode of an escalator. Not every incoming agent has directly access to the escalator since the required space in the entering region is occupied by other agents. As a consequence, a congestion is forming in front of the facility.

In all simulation aiming to investigate the capacity we therefore ensure that the second condition is fulfilled. As long as this is the case, the actual value of the ration α/β does *not* affect the value of the obtained capacity which we checked explicitly.

In order to avoid possible misunderstanding, we added the following sentence to the manuscript: *"Only with this condition it is guaranteed that the escalator is operated in a mode that corresponds to its capacity."*

Point 2 : In section "Speed dependence of the time gap" the authors write "Regarding in particular capacity values stated in [3, 5, 19], however, good agreement is achieved by using $A5 = 0.6s^6m^{-5}$ and $Aj = 0$ for the other coefficients". Do the authors have any clues as to why there is no low-order dependence of the time gap on speed?

Response 2 : If one aims to describe a *decrease* of the escalator capacity for larger conveyor speeds (which by no means is consensus even among manufactures), speed-dependent terms can be added to T as done in Eq. (18). These additional terms can be interpreted as human 'hesitation' time that is related to the entering process of the passengers. In contrast to the human reaction time, the hesitation can have a vanishing value.

In general, hesitation can be assumed to have a strong velocity-dependence since passengers are more likely to hesitate when entering a fast moving escalator than a slower one (In principle, another non-linear relationship, even a step function or a threshold, is conceivable). According to this, the reason why there is no low-order dependence of the time gap when using a power series is that low-order terms would distort the capacity results that are obtained for lower conveyor speeds. These terms, however, can be assumed to not have a large impact from hesitation, as mentioned before and, more importantly, they are also in good agreement with experimental data and data provided by manufacturers, as discussed below Eq. (16).

The authors are aware of the fact that there is no "physical" justification for the additional hesitation terms in Eq. (18) due to the lack of corresponding experimental data and due to contradictory information of manufacturers. We therefore write explicitly: "The implementation of speed-dependent terms in Eq. (18) is by no means unique since the underlying velocity-dependence is (so far) unknown. Using a power series is only *one possible* realization."

We also agree that in the current form of the manuscript, Eq. (18) could be erroneously interpreted as the main result of the paper. Actually, however, this extension mainly concerns high escalator speeds. In order to highlight that, from our point of view, Eq. (16) is in fact more important, we moved the section discussing the speed dependence of the time gap to the appendix and slightly modified the section title to "*Possible* speed dependence of the time gap".

Point 3 : In the conclusion, the authors write "In this way, the deduced capacity formula delivers a solid basis to ease the decision-making of operators to find the optimal conveyor speed that is simultaneously compatible with safety, comfort, and performance demands." How do the authors believe that "safety" and "comfort" could be estimated from the simulations?

Response 3 : Finding the "optimal" conveyor speed indeed includes to balance out (at least) the three major aspects performance, safety, and comfort. In our contribution, we aim to contribute in facilitating this process by focusing on *one particular* of these points, namely on the performance aspect. We do this by developing an analytical formula quantifying the handling capacity of escalators. The other two aspects are, of course, not described by the formula. We therefore agree that the above-mentioned sentence could be misinterpreted and therefore modified it accordingly: *"In this way, the deduced capacity formula delivers a solid basis for the performance analysis of escalators and contributes to the decision-making of operators to find the optimal conveyor speed."* Accordingly, we also slightly modified the introduction to avoid any confusion. For example, we now write *"In the following, we aim to contribute to resolving this issue by putting the speed-capacity relation and therefore the performance analysis of escalators on a firmer basis."*

Response to the comments of reviewer 2

Dear reviewer,

we would like to thank you for your efforts in studying our article, and your useful suggestions, which contribute to improve our work. Please find below our answers to each of your comments and a description of the corresponding modifications done in the manuscript.

The manuscript "Come together: A unified description of the escalator capacity" by Christoph Gnendiger and colleagues model passengers dynamics on escalators. The authors model this system using an agent model and obtain an analytical formula for the escalator capacity as a function of the escalator velocity and a T parameter that they interpret as a human reaction time scale. In general the paper is interesting, however, I have some reservations about a couple of things.

Point 1 : My main issue with the manuscript is that the main finding is the fact that the model agrees with data when using T between $0.15s$ and $0.30s$, which then they conclude to be in agreement with human reaction time from social psychology experiments. However, this is misleading. The model only agrees with the experiments when they artificially expand T as a polynomial function of v_{esc}. The original model does not agree with the data. They use the 5th power on this expansion without any physical justification. By doing this they are basically fiting C_{esc} as an inverse power series of v_{esc}. The authors must make this very clear along the text and abstract.

Response 1 : We agree with the fact that the interpretation of the (initially unphysical) model parameter T as human reaction time is not directly obvious. We also agree that the way we presented the results so far may give the impression that Eq. (18) is actually more important than Eq. (16). However, the opposite is true. The authors believe that Eq. (16) is one of the most important findings and we mention this several times in the text, including the conclusions. Moreover, the non-linear extension of the time gap in Eq. (18) essentially concerns only large escalator speeds. In this respect, we would like to clearly state the following points:

- Since we are investigating the handling capacity of an escalator, which by definition is *larger than* (or at least as large as) any observed flow, Eq. (16) is compatible with *all* observations made in field experiments and with data provided by manufacturers. This can be seen, for instance, in Fig. 9 in which predictions of Eq. (16) are compared with empirical data. The goal of the comparison is not to *fit/connect* data points of a particular field study or a particular manufacturer. Instead, the goal is to find a function that is *larger* (or at least as large as) any data point in Table 1. And indeed, the solid blue line lies above any observed value, as it should be.

- Second, the comparison clearly shows that it is indeed possible to narrow the range of possible T values such that it is compatible with the state of knowledge of human/mental chronometry and social psychology. For instance, we write explicitly "The minimum value of the time gap to explain *all* results in Table 1 is $T = 0.15\,s$." and "A time gap of $T = 0.35\,s$ is too long to be compatible with observed flows of [12, 14–16] and data provided by [3–5]. In order to explain these experimental data with Eq. (16), therefore smaller T-values have to be used ...". All these statements refer to the original model quantified by Eq. (16) *without* replacement (18).

- Only if one aims to describe a *decrease* of the escalator capacity for larger v_{esc} (which by no means is consensus even among manufactures as stated in the text), speed-dependent terms can be *added* to T as done in Eq. (18). In this respect, Eq. (18) is not an expansion of T but only an *extension*. In principle, another non-linear relationship, even a step function or a threshold, is conceivable.

- The authors are aware of the fact that there is no "physical" justification for this extension due to the lack of corresponding experimental data and due to contradictory information of manufacturers. We therefore write explicitly: "The implementation of speed-dependent terms in Eq. (18) is by no means unique since the underlying velocity-dependence is (so far) unknown. Using a power series is only *one possible* realization." Moreover, in the conclusions we write: "Also regarding the speed-dependence of the time gap, only first considerations have been conducted in the present contribution. These aspects as well as their impact on the escalator capacity should be carefully integrated into future work."

Summing up, we agree that in the current form of the manuscript, Eq. (18) could erroneously be interpreted as the main result of the paper. In order to highlight that, from our point of view, Eq. (16) is in fact more important, we moved the section discussing the speed dependence of the time gap to the appendix and slightly modified the section title to "*Possible* speed dependence of the time gap".

Apart from that, I have some small comments:

Point 2 : Table 1 is not totally clear for me. The authors refer to "clear width" and here I was lost, because only much later they introduce this parameter. I think they must explain this before, since they will write about this at Table 1. Also, what is "count interval"?

Response 2 : Regarding the definition of the clear width w, we agree with the comment of the reviewer and changed the caption of Tab. 1 accordingly. Moreover, we now explain the definition of 'time interval' in the caption: "The column 'count interval' indicates the length of the time span in which the maximum flow is measured."

Point 3 : In the end of the section "Model of pedestrians", the authors define a set of parameters, $a_{agent} = a_{wall} \ldots$ without any proper explanation. I suppose it is something related with an interaction with the wall, but how? What are the D's? What are the physical unit of the a's?

Response 3 : The author's intention behind the explicit listing of the used model parameters is not to support the argumentation in the text. The parameters are given only for the sake of completeness, allowing potential users of the software JuPedSim to reproduce the simulation results. The parameters itself and their definition are therefore not explained in detail as this constitutes a potential source of distraction from the main line of argumentation. Instead, the authors explicitly refer to the public manual of the simulation software and to the original paper of the operational model: "In this work, we use default values for the model parameters as suggested in [21,26,29]: ...".

Nevertheless, however, in the manuscript we now provide details and physical interpretations of the pedestrian model parameters: *"Here, a_{agent} and a_{wall} are dimensionless repulsion coefficients while D_{agent} and D_{wall} are repulsion distance thresholds between pedestrians and with the geometry, respectively."* Parameter a has no physical unit, as given in the text.

Point 4 : On section "Model of escalator" page 5. The authors claim: "Comparing the requirements with Fig. 3, it follows that the deceleration and acceleration of the agent can be adjusted such that it takes place fast enough." I do not know if this is totally clear. For me this is a strong claim. What led you to claim that? The math being right does not imply that is possible in reality. Perhaps, the authors can clarify this statement.

Response 4 : The values in Tab. 2 are not obtained by the authors of the present contribution but they have been *fixed*, as stated in the text, by the international norm EN 115-1. In this norm, for instance, it is explicitly stated that in case an escalator is operated at $v_{esc} = 0.5\,m/s$, the horizontal flights at the entrance and the exit areas need to have a length of *at least* $0.8\,m$, respectively. When we obtain an adaptation length of $\approx 0.4\,m$ from the simulations, as in the right panel of Fig. 3, we are convinced that it is indeed justified to state that the simulation results are compatible with the requirements of norm EN 115-1.

Point 5 : Reading pag. 7, after Eq. (3), I am not sure if I fully understand the dynamics. Did you define "y" at some previous point? How is the dynamics on y? I think the model in general is not clear regarding the dynamics.

Response 5 : Similar to x_j (which is the absolute x-position of agent j), y_j is defined as the absolute y-position of that agent. Referring the Eq. (3), this was so far only mentioned in the text. In order to avoid any misunderstanding, we now explicitly provide the definition of Δy in Eq. (3b).

Point 6 : I can not see all the curves in Fig. 5. Are they overlapping? Also, what it is s? Was it properly defined at some point?

Response 6 : Since the average distance in y-direction, Δy, almost vanishes in the upper two diagrams of Fig. 5 (which can be seen in the legend), the total distance s is approximately the same as Δx. The corresponding curves are therefore overlapping. In order to avoid any confusion, we added the following sentence to the caption of Fig.(5): *"Since Δy almost vanishes in the upper two diagrams, the total distance s is approximately the same as Δx."*

Similar to Δx and Δy (which are average distances in x- and y-direction, respectively), s is the average of the *total* distance between the agents. Referring the Eq. (3), this was so far only mentioned in the text. In order to avoid any misunderstanding, we now explicitly provide the definition of s in Eq. (3c).

Point 7 : In general for me it was confusing if I was looking at a continuous model or a discrete one due to the nature of escalators. I think the paper can improve if the authors could separe this better and make clear how the results depend on these two possible conditions.

Response 7 : We agree that escalator-related quantities like the distance in movement direction or the step occupancy are initially discretized due to the presence of individual escalator steps. The point is, that in the curse of investigation, we do not investigate these quantities directly, but consider their *average* of an extended period of time, see for instance Fig. 5. Therefore, average distances, occupancies, flows, and capacities are no discrete but (quasi) continuous quantities. Therefore, the (space-)continuous model presented in the contribution is capable of adequately describing the passenger dynamics of escalators.
